# Shading affects the nitrogen cycling process and plant nitrogen uptake by altering the rhizosphere microbial community

**DOI:** 10.3389/fpls.2026.1780344

**Published:** 2026-04-02

**Authors:** Guoyi Yuan, Xuanquan Zhu, Liuchen Zhang, Xuejian Wang, Yihui Wang, Donghai Guo, Tikun Zhang, Ge Wang, Na Wang

**Affiliations:** 1 College of Resources and Environment, Yunnan Agricultural University, Kunming, China; 2 College of Tobacco Science, Yunnan Agricultural University, Kunming, Yunnan, China; 3 Pu ‘er Branch of Yunnan Provincial Tobacco Company, Pu ‘er, Yunnan, China; 4 China Tobacco Shandong Industrial Co., Ltd., Jinan, Shandong, China

**Keywords:** amino acid, nitrogen cycle, rhizosphere soil, shading, tobacco

## Abstract

Plants adapt to environmental changes by affecting the rhizosphere environment and microbial pathways. Shading affects nitrogen absorption and accumulation in plants by directly or indirectly altering the light intensity. However, the effects this has on the rhizosphere micro-environment and especially the microbial community are not fully understood. Utilizing non-targeted metabolomics and metagenomics, we investigated the changes in the microbial community structure in the cigar tobacco rhizosphere and the nitrogen cycling process and its relationship with nitrogen absorption by the plants under artificial shading conditions. Shading significantly increased the rhizosphere soil organic carbon, hydrolyzable nitrogen, ammonium nitrogen, nitrate nitrogen, and nitrogen contents in tobacco plants. Metabolomics revealed that shading significantly affected the arginine biosynthesis pathway in the rhizosphere soil, with the expression levels of L-oxornithine, citrulline and L-arginine significantly increasing. Metagenomics analysis indicated that shading significantly altered the rhizosphere microbial community structure and the nitrogen cycling process. The abundances of organic nitrogen-decomposition (*gdh A*, *ansB*) and nitrification genes (*amoA_B, amoB_B, amoC_B, hao*) significantly increased. *Flavobacterium* and *Stenotrophomonas* may play important roles in the nitrogen cycle in the rhizosphere. Correlation analysis indicated that *Flavobacterium* and *Stenotrophomonas* were significantly positively correlated with L-glutamic acid, L-ornithine and L-arginine (*p* < 0.05). These results reveal the biological mechanism by which shading affects nitrogen absorption in crops via changes in the rhizosphere microbial community and the nitrogen cycling process, providing a scientific foundation for guiding nutrient management strategies in shaded cultivation.

## 1 Introduction

The close connection between plants and the soil is achieved through plants’ adaptation to environmental changes and the release of root exudates into the soil, which shapes the rhizosphere environment and microbial pathways (Hu L. et al., 2018; Korenblum et al., 2022). This process maintains soil element cycling and promotes nutrient absorption by the roots (Liao et al., 2024; Xiao et al., 2024). Plants exude 20–40% of their photosynthetic products as secretions through the roots into the rhizosphere, shaping a rhizosphere environment that significantly differs from the bulk soil (Zhalnina et al., 2018; Canarini et al., 2019; Prescott et al., 2020; Park et al., 2023). As a key factor influencing plant photosynthesis, changes in light intensity affect the expression of metabolites exuded from the plant into the rhizosphere, thereby altering the rhizosphere micro-environment, especially the structure and function of the microbial community. The rhizosphere microenvironment is the most critical area for the interaction between plants and microorganisms, and it affects nutrient absorption and plant growth and development.

Nitrogen (N), known as the “element of life”, is a crucial factor limiting plant growth, development, yield, and quality. The N acquisition strategies of plants depend on biological and non-biological factors, such as plant functional types, soil, and climatic conditions (Dai et al., 2014; Zhang et al., 2018; Freschet et al., 2021). Light plays an important role in influencing and regulating N absorption and utilization in plants (Koleszar et al., 2022; Zhao et al., 2024; de Silva et al., 2025; Xu et al., 2025). Previous studies have mainly focused on the changes in related physiological and biochemical processes in plants that are mediated by light and the regulatory mechanisms associated with these changes on N assimilation and transport. However, little is known about the pathways and mechanisms through which changes in light intensity indirectly regulate the micro-environment in the plant–soil system and thereby affect N absorption by plants.

N availability at the root–soil interface in the plant–soil system is of vital importance for plant N acquisition. The rhizosphere microbial community can transform complex organic compounds into NH4^+^-N and NO3^−^-N, which are easily absorbed by crops, driving the decomposition of soil organic matter, the conversion of inorganic substances, and interactions with other organisms (Maia and Moura, 2014; Pizarro-Tobias et al., 2020). This plays a core role in driving soil N cycling and regulating the direction and intensity of the cycling process (Nelson et al., 2016; Sahu et al., 2017; Fang et al., 2025). For instance, in the corn–soybean intercropping system, the expression of nrfC and nirA genes in the microbial community were significantly upregulated, thus promoting N fixation and transfer (Dong et al., 2024). In rice, zinc fertilizer application alters the microbial community structure in the rhizosphere soil and upregulates the expression of denitrification genes (*napA* and *nirS*), accelerating N transformation and improving the N utilization rate (Lv et al., 2022). However, it remains unclear how shading affects the microbial community structure, especially functional genes related to N cycling.

Shade cultivation is widely regarded as a sustainable agricultural practice that improves crop growth environments and enhances product quality. It has been extensively adopted in diverse regions (Chen et al., 2021a). Scientific evidence indicates that reasonable shade can modulate coffee berry yield and ripening (Hao et al., 2022), enhance the flavor and aroma profile of tea (Niu et al., 2025), and increase the content of bioactive compounds in various medicinal plants (Xu et al., 2024; Gao et al., 2025; Zhu et al., 2026). The cigar wrapper leaf, as a key component, directly determines cigar quality (Zhang et al., 2021). Cigar wrapper leaves are cultivated under shade to achieve better physical and chemical properties (Ma et al., 2023; Yan et al., 2024). However, the mechanisms by which artificially altering light conditions regulate the micro-ecological characteristics of the rhizosphere soil and N absorption in tobacco plants remain unclear. We hypothesize that shade-cultivated cigar tobacco alters the interactions between metabolites in the rhizosphere of tobacco plants and the microbial community and directly or indirectly affects the N acquisition of the plants. Therefore, this study employed non-targeted metabolomics and metagenomics to investigate the rhizosphere metabolic characteristics of cigar tobacco after shading and the microbial mechanism through which it indirectly affects the N cycle by altering the microorganisms. The main objectives of this study were as follows: (1) to determine the effects of shading on rhizosphere metabolites; (2) to identify the changes in the rhizosphere microbial community after shading, especially the influence of N cycle-related functional genes and key groups; and (3) to preliminarily clarify the intrinsic relationships among shading, rhizosphere metabolites, the key groups, and the N cycle. The results of this study provide a theoretical basis for efficient nutrient utilization in crops cultivated under shading, enabling sustainable agricultural production.

## 2 Materials and method

### 2.1 Test materials

The tested tobacco (*Nicotiana tabacum* L.) variety was ‘Yunxue No.36’(Yunnan Academy of Tobacco Agricultural Sciences, Yuxi, China), and the fertilizer used in this study was compound fertilizer (N:P_2_O_5_:K_2_O = 12:12:24, total nutrient content ≥ 48%) (Yunye Fertilizer Co., Ltd., Kunming, Yunnan, China). The shading shed was constructed using steel frames, with the shading net 3 m away from the bottom of the ridge. The shading material was a white high-density polyethylene mesh, with a nominal light transmittance of approximately 70% (the shading rate is about 30%).

### 2.2 Experimental design

This experiment was conducted from April to June 2023 in the cigar tobacco production area of Ninger County, Puer City, Yunnan Province (latitude 23°9’0”N, longitude 101°6’36”E, altitude 860 m). The annual average temperature, sunshine duration, and precipitation in this area were 18.7°C, 1921 h, and 2283 mm, respectively, and according to the “Soil Classification Standard of the People’s Republic of China” (GB/T 17296-2009), the soil type of this test site is described as red soil. The physical and chemical properties of the soil were as follows: pH 6.31; hydrolyzable nitrogen (HN), 58.32 mg/kg; soil organic carbon (SOC), 11.25 g/kg; available phosphorus (AP), 17.36 mg/kg; available potassium (AK), 285 mg/kg; ammonium nitrogen (NH4^+^-N), 4.16 mg/kg; and nitrate nitrogen (NO3^−^-N), 7.67 mg/kg.

The shaded area (S) was designated as the treatment group, and the unshaded area (NS) served was the control group. Each experimental group comprised a total area of 616 m², which was evenly subdivided into eight plots that functioned as sampling points. At 60 days post-transplantation, corresponding to the tobacco maturity stage, three tobacco plants exhibiting consistent growth, along with their associated rhizosphere soil samples, were randomly collected from each plot. The three plant samples and the three soil samples were then pooled separately to constitute one biological replicate. Each experimental group thus yielded a total of eight biological replicates.

There were 200 tobacco plants in each plot, and the plant spacing was 35 cm × 110 cm. Tobacco was transplanted on April 30. According to the methods of previous researchers (Tao et al., 2024), cigar tobacco was uniformly propagated through seedling cultivation and was transplanted when the seedlings had 5 or 6 leaves. Before transplant, 492 kg ha^−^¹ of compound fertilizer was applied as a base fertilizer. At 25 days after transplant, an additional 328 kg ha^−^¹ of the same compound fertilizer was applied with irrigation as top dressing. The total nitrogen input was 98.4 kg N ha^−^¹. With the exception of whether plots were shaded or not, all other agricultural operations in each experimental group were consistent.

### 2.3 Measurement of light parameters

To precisely quantify the light environment created by the shading treatment, both photosynthetically active radiation (PAR) and spectral composition were measured *in situ*. Measurements were taken at the top of the plant canopy during the solar noon period (12:00–13:00 local time) on a typical sunny day within the key experimental growth phase, using a spectroradiometer (SPIC-300AW, Everfine, China).Within each plot, the sensor was placed horizontally at the canopy level at five randomly selected points.

The Photosynthetic Photon Flux Density (PPFD) was 1754.9 μmol m^−^² s^−^¹ in the control (NS) and 1220.3 μmol m^−^² s^−^¹ under the shading treatment (S). Thus, the light intensity under shading was approximately 70% of the control, which aligns with the nominal transmittance of the shading net. Concurrently, the shading net significantly altered light quality, reducing the red to far-red ratio (R:FR) at the canopy level from 1.3211 (NS) to 1.2857 (S). For details, see **Supplementary Table S1**.

### 2.4 Net photosynthetic rate, fresh and dry weight, and nitrogen content of leaves

Prior to sample collection, the net photosynthetic rate (Pn) of the selected plants was measured from 9:00 to 11:00 using a LI-6400 XT portable photosynthesis system (LI-COR 6400, LICOR Inc., Lincoln, NE, USA). The measurement conditions were set as follows: leaf chamber temperature at 25 °C, air flow rate at 500 µmol·s^−^¹, CO_2_ concentration at 400 µmol·mol^−^¹, and photosynthetic photon flux density at 1500 µmol·m^−^²·s^−^¹.

All roots, stems, and leaves of selected tobacco plants were collected, washed with clean water, and dried with sterile paper. The fresh weights of the roots, stems, and leaves were determined separately. (JWE I, JADEVER, Shenzhen, China). The roots, stems, and leaves of each tobacco plant were placed in an oven (ED-S 56, Binder, Germany), dried at 105°C for 30 min, and then dried at 75°C for 72 h. The dry weights of the roots, stems, and leaves of the tobacco plants were determined separately, and the root-to-shoot ratio was recorded. After drying, the root, stem, and leaf samples were ground and passed through a 0.5-mm sieve. The H_2_SO_4_-H_2_O_2_ method was used to digest the samples until they became clear. The total N content in the roots, stems, and leaves was then determined separately using a flow analysis instrument (AA3, Seal Analytical Ltd., Rugby, UK) (Hu et al., 2021).

To quantify nitrogen partitioning among different organs, we calculated the Nitrogen Allocation (NA) index, which represents the proportion of total plant nitrogen accumulated in the roots, stems, and leaves (Hu F. et al., 2018). The calculation formula is:


Nai (%)=(Naci/Nact)×100

where, Nac_i_ and Nac_t_ represent the nitrogen concentration in a specific plant organ (leaf, stem, or root) and the total plant nitrogen concentration, respectively. Nact is calculated as the sum of nitrogen accumulated across all organs.

### 2.5 Leaf chlorophyll content

After the determination of the leaf photosynthetic parameters, samples were collected from the 12th leaf (from the bottom to the top) of the tobacco plants. The leaf tissue (0.1 g fresh mass, n = 8) was homogenized in 1.5 mL of 95% methanol. Samples were centrifuged at 9000×g for 5 min, and the supernatant was collected. For plastid pigment content determination, the absorbance was measured using a spectrophotometer (SHIMADZU-UV 2600 spectrophotometer, Shimadzu Corporation, Kyoto, Japan). The chlorophyll a and b contents were measured using spectrophotometry at 665 and 649 nm wavelengths (Lichtenthaler and Wellburn, 1983). Total chlorophyll was calculated using the following equation: total chlorophyll= chlorophyll a + chlorophyll b.

### 2.6 Soil physical and chemical properties

The root systems were gently shaken to remove the loose soil, and soil adhering to the root systems was collected using a soft-bristled brush as the rhizosphere soil. This soil was sieved through a 2-mm aperture. Each soil sample was divided into two parts. One part was immediately used to determine the soil physical and chemical properties, and the other was flash frozen in liquid N and stored at −80 °C for DNA extraction and liquid chromatography–tandem mass spectrometry (LC-MS/MS) (Waters Corporation, Milford, MA, USA) analysis of metabolites.

The soil pH was measured using an E20-FiveEasy pH meter (Mettler Toledo, Giessen, Germany); the soil organic carbon (SOC) was determined using a total organic carbon/total nitrogen (TOC/TN) analyzer (multi N/C2100S, Analytik Jena AG, Germany) (Zhang et al., 2025). The NH4^+^-N and NO3^−^-N in the soil were determined using the indophenol blue spectrophotometric method (Zhan et al., 2024) and the ultraviolet spectrophotometric method (UV-1601, Shimadzu Corporation, Kyoto, Japan) (Wang et al., 2024), respectively. The soil HN was determined using the alkali hydrolysis reduction diffusion method (Liu et al., 2020).

### 2.7 Untargeted metabolomics of rhizosphere soil

A 1-g rhizosphere soil sample was ground with liquid nitrogen, mixed with 1000 µL 80% methanol water solution, and vortexed. The sample was placed on ice for 5 min and centrifuged at 15, 000 rpm for 15 min at 4°C. The supernatant was collected and centrifuged again at 15, 000 rpm for 20 min at 4°C. The supernatant was freeze-dried, mixed with 10% methanol solution, and injected into the liquid chromatography–mass spectrometry (LC-MS) system (Waters Corporation, Milford, MA, USA) for analysis.

The sample was injected onto an ACQUITY UPLC BEH Amide Column (HILIC) (Waters Corporation, Milford, MA, USA) at a flow rate of 0.2 mL/min for 17 min, with a linear gradient. Mobile phase A was 90% acetonitrile and 5 mM ammonium acetate; mobile phase B was 50% acetonitrile and 5 mM ammonium acetate. The chromatographic gradient elution program was performed as follows: 2% B for 0 min, 2% B for 1.5 min, 100% B for 7 min, 100% B for 9 min, 2% B for 9.1 min, 2% B for 10 min, and 2% B for 12 min. The mass spectrometry conditions were set as follows: spray voltage, 3.5 kV; sheath gas flow rate, 35 psi; auxiliary gas flow rate, 10 L/min; capillary temperature, 320 °C; s-lens RF level, 60; auxiliary gas heater temperature, 350 °C; polarity, positive and negative; and MS/MS secondary scans, data-dependent scans.

The off-machine data were subjected to peak extraction, peak quantification, and peak alignment using XCMS (version 4.0.2). Metabolite identification was based on a 10 ppm mass deviation and information, such as adduct ions, and by comparison with the high-quality secondary spectral database (NovoMetDB) established by Novogene Co., Ltd. (Beijing, China). The identified metabolites were annotated using the Kyoto Encyclopedia of Genes and Genomes (KEGG) database (https://www.genome.jp/kegg/pathway.html).

### 2.8 Metagenomics sequencing

Total DNA was extracted from 0.5 g of homogenized soil from each sample using a Fast DNA^®^SPIN Kit for Soil (MP Biomedical, Santa Ana, California, USA). The concentration of the extracted DNA was measured using a Qubit 4.0 fluorometer (Invitrogen, USA). The soil DNA purity was evaluated using a NanoDrop 2000 spectrophotometer (Thermo Scientific, USA). Approximately 1 μg of qualified DNA was collected from each sample and sent to Novogene Co., Ltd. (Beijing, China) to construct the sequencing library.

The raw data were subjected to quality control using fastp (version 0.20.0, https://github.com/OpenGene/fastp). High-quality reads were assembled using MEGAHIT software (version 1.1.2), and the resulting scaffolds were fragmented to obtain scaffolds without N bases. MetaGeneMark software (http://topaz.gatech.edu/GeneMark/) was used to predict the open reading frames (ORFs) for the scaffolds (≥500 bp) of each sample. ORFs with a length of more than 100 bp were retained. CD-HIT software (version 4.6.1, http://www.bioinformatics.org/cd-hit/) was employed to remove redundancies and obtain a non-redundant initial gene catalogue. Using Bowtie2 (V2.5.4), the clean data were aligned to the initial gene catalogue, and the number of reads that each gene matched in each sample was calculated. Genes with a read count of ≤2 in any sample were filtered out. The final gene catalogue (unigenes) was obtained for subsequent analysis. The unigenes were searched and aligned in the Micro NR and NCycleDB databases. Species annotation and gene function identification were performed using DIAMOND software (version 0.8.35).

### 2.9 Data analysis and visualization

Using Graph Pad Prism (version 10.4.1), independent sample t-tests and visualization were conducted for the net photosynthetic rate, fresh and dry weight, chlorophyll content, N content of tobacco plants, soil physical and chemical properties in the rhizosphere, and relative abundance of N cycle-related functional genes. Using the ‘tidyverse’, ‘microeco’, and ‘magrittr’ packages in R (version 4.4.1), linear discriminant analysis (LDA) effect size (LEfSe) analysis (with LDA > 3.0) was conducted on the soil microbial community data, and the results were visualized using the ‘plot_diff_bar’ function. The free online data analysis platform OmicShare (https://www.omicshare.com/) was used to conduct species contribution analysis of key genes in the N cycle and Pearson correlation analysis and visualization of differential metabolites (DMAs) and related species in the N cycle. The Metware Cloud Online Analysis Platform (https://cloud.metware.cn) was used to conduct principal component analysis (PCA), DMA screening (variable importance in projection (VIP) ≥ 1, p < 0.05, |log_2_(fold change)| ≥ 0.58), and KEGG enrichment analysis.

## 3 Results

### 3.1 Influence of shading on the photosynthetic pigments of tobacco plants and their growth

**Figure 1:**
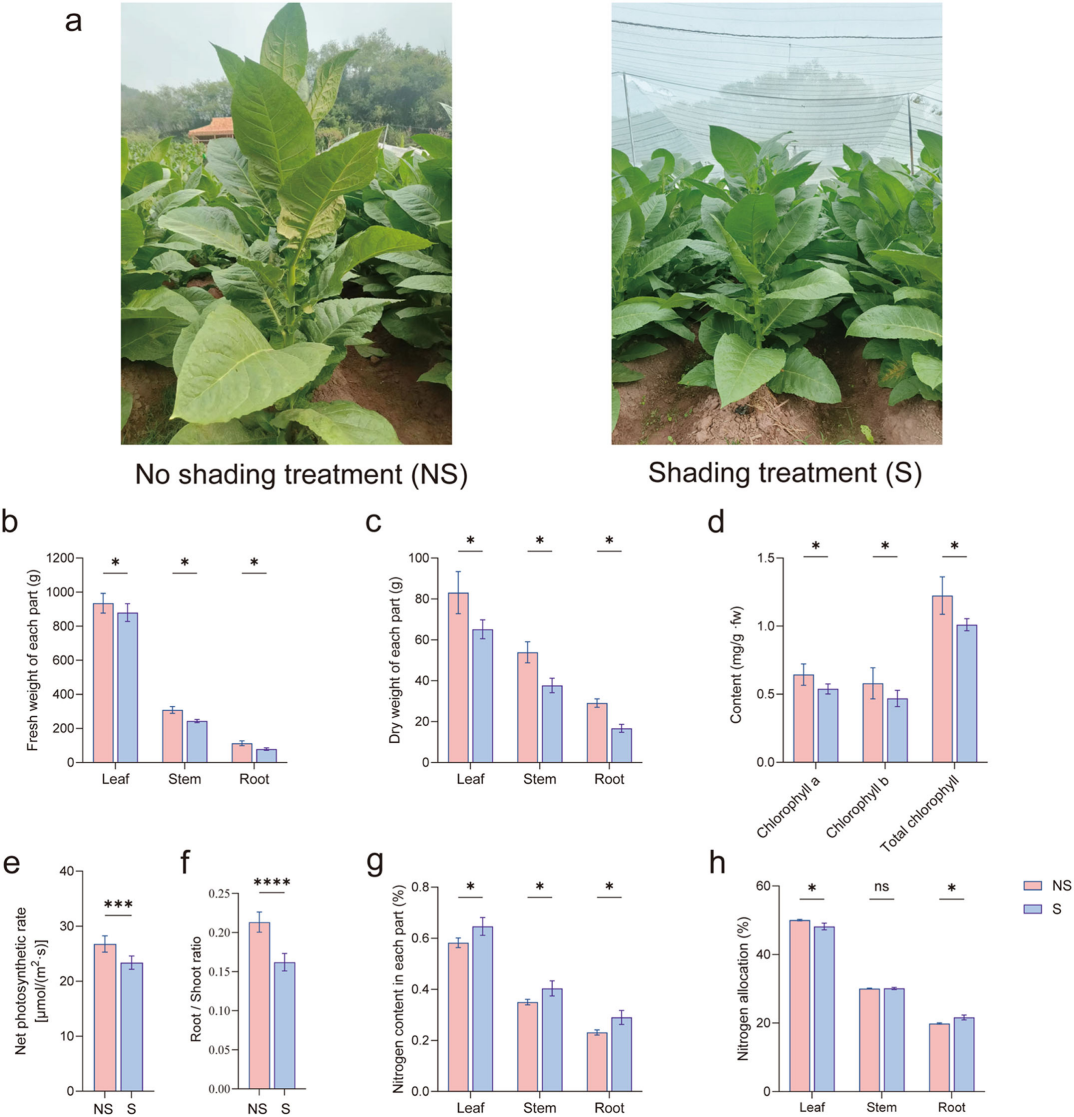
Growth and physiological indicators of shaded (W) and non-shaded (M) tobacco plants. **(a)** Growth and appearance of the tobacco plants. **(b)** Fresh weight of each organ of the tobacco plant. **(c)** Dry weight of each organ of the tobacco plant. **(d)** Contents of chlorophyll a, chlorophyll b, and total chlorophyll. **(e)** Net photosynthetic rate of the leaves. **(f)** The root-shoot ratio of tobacco plants. **(g)** Nitrogen content in each part. **(h)** Nitrogen allocation ratios in tobacco plant organs. Statistically significant differences are indicated by asterisks (t-test, n = 8): * (*p* < 0.05), *** (0.001 < *p* < 0.002), **** (*p* < 0.001), 'ns' (not significant). The data are presented as the mean ± standard error.

Shading had a significant impact on the growth and appearance of tobacco plants (**Figure 1a**). Compared with the control, shading significantly reduced leaf, stem, and root fresh weights by 5.9%, 21.03%, and 29.93%, respectively; the corresponding dry weights were reduced by 21.60%, 30.20%, and 42.66% (**Figures 1b, c**). Meanwhile, shading significantly inhibited chlorophyll synthesis in leaf plastids, reduced the net photosynthetic rate (by 12.67%), and also lowered the root-to-shoot ratio (by 24.01%) of the tobacco plants (**Figures 1d–f**). However, shading significantly increased the nitrogen content in leaves (by 10.64%), stems (by 15.36%), and roots (by 25.62%) (**Figure 1g**). Concurrently, the proportion of nitrogen allocated to roots increased by 9.01%, while it decreased in leaves by 3.72% (**Figure 1h**). These results indicate that shading impairs photosynthetic carbon assimilation and inhibits growth in tobacco. Under the resulting carbon-limiting conditions, tobacco plants optimize survival by increasing nitrogen allocation to the roots, thereby enhancing their adaptation to limited light.

### 3.2 Effects of shading on the physical and chemical properties and metabolic characteristics of rhizosphere soil

**Figure 2:**
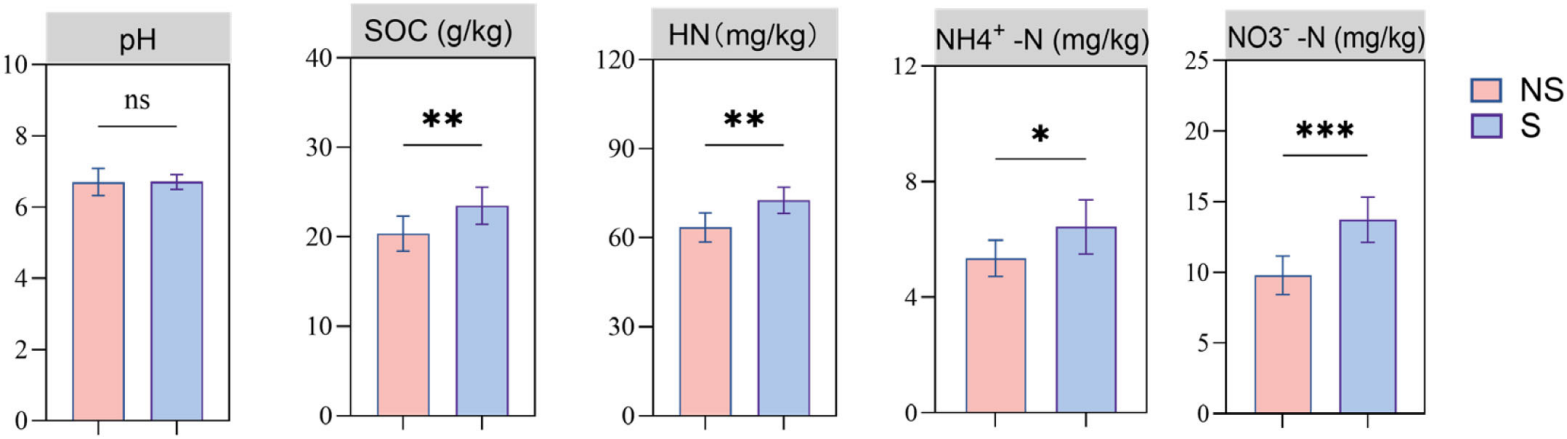
Physical and chemical properties of the rhizosphere soil of shaded (S) and non-shaded (NS) tobacco plants. SOC, organic carbon; HN, hydrolyzed nitrogen; NH_4_^+^-N, ammonium nitrogen; NO_3_^−^-N, nitrate nitrogen. Statistically significant differences are indicated by asterisks (t-test, n = 8): * (p < 0.05), ** (p < 0.01), *** (p < 0.001) and ‘ns’ (not significant). Data are presented as the mean ± standard error.

After shading, the SOC, HN, NH_4_^+^-N, and NO_3_^−^-N contents in the rhizosphere soil of the tobacco plants increased significantly by 12.02, 12.51, 16.83, and 28.68%, respectively, indicating that shading had a significant impact on soil N (**Figure 2**).

**Figure 3:**
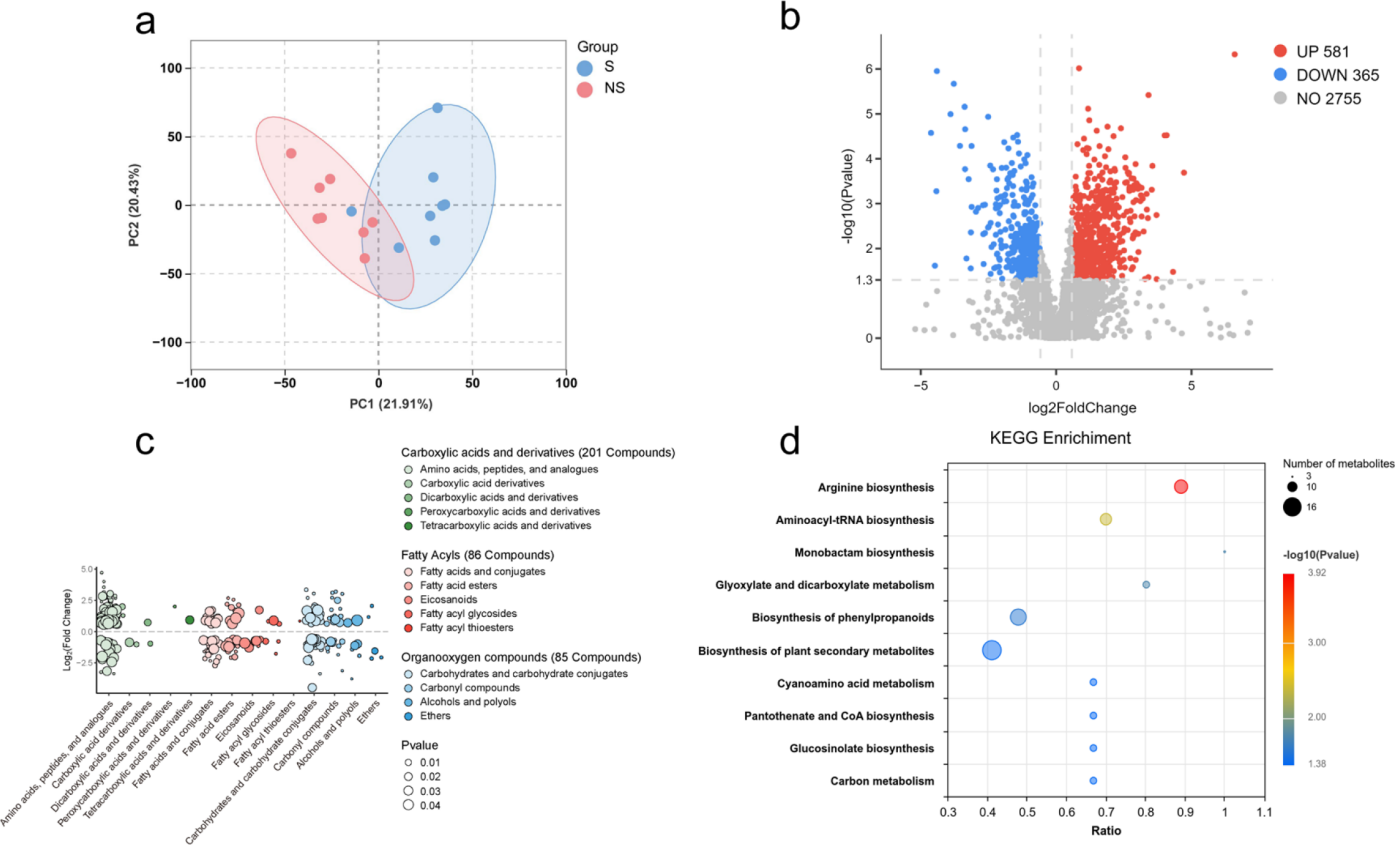
Metabolomics analysis of rhizosphere soil under shading (S) and non-shading (NS) conditions. **(a)** principal component analysis of S_vs_NS. **(b)** Number of upregulated and downregulated differential metabolites in S_vs_NS. **(c)** Classification screening of differential metabolites in S_vs_NS in the second-level category (top 3 in quantity) and the third-level category (top 5 in quantity). **(d)** Top 10 KEGG enrichment pathways of differential metabolites (n = 8).

Untargeted metabolomic analysis was performed on tobacco rhizosphere soil samples. After quality control, a total of 3, 691 metabolites were retained and classified into 16 categories (**Supplementary Table S2**). These metabolites were successfully detected and quantified in all samples and were used for subsequent analyses. Among them, lipids and lipid-like molecules accounted for the highest proportion (24.44%), followed by organic acids and derivatives (23.79%) and organoheterocyclic compounds (18.50%). The Pearson correlation coefficient between quality control (QC) samples was calculated based on the relative quantitative values of metabolites. The correlation coefficient |r| for all QC samples was ≥0.993, indicating good detection stability and high data quality (**Supplementary Figure S1**). PCA showed that shading significantly affected the metabolism of the rhizosphere soil. PC1 and PC2 accounted for 21.91 and 20.43% of the variance, respectively (**Figure 3a**). A volcano plot revealed the screening results for the DMAs in the S_vs_NS comparison group (*p* ≤ 0.05, VIP ≥ 1, |log_2_(fold change)| ≥ 0.58). A total of 946 DMAs were screened, among which 581 were upregulated and 365 DMAs were downregulated (**Figure 3b**). Specific information on the DMAs can be found in **Supplementary Table S3**.

To provide a detailed overview of the metabolite composition, we classified the identified metabolites into three levels. The first-level category represents superclass (e.g., Lipids and lipid-like molecules), the second-level category represents class (e.g., Fatty Acyls), and the third-level category represents subclass (e.g., Amino acids, peptides, and analogues). We re-filtered all obtained DMAs (**Figure 3c**) and sorted them by category. We then selected the top 3 DMAs in the second-level category and the top 5 DMAs in the third-level category, as shown in **Supplementary Table S4**. Among the 946 DMAs, the number of carboxylic acids and their derivatives was the largest (201), followed by fatty acyls (86) and the number of organooxygen compounds (85). Among the 201 carboxylic acids and derivatives, the substances with the most significant upregulation and downregulation were amino acids, peptides, and analogues. KEGG pathway enrichment analysis was conducted on DMAs to determine the differences in metabolic pathways in the S_vs_NS comparison group (**Figure 3d**), and the Arginine biosynthesis pathway had the highest enrichment degree (**Supplementary Table S5**). Therefore, we chose this pathway as the key pathway for further in-depth study of the root–soil metabolic response to shading.

**Figure 4:**
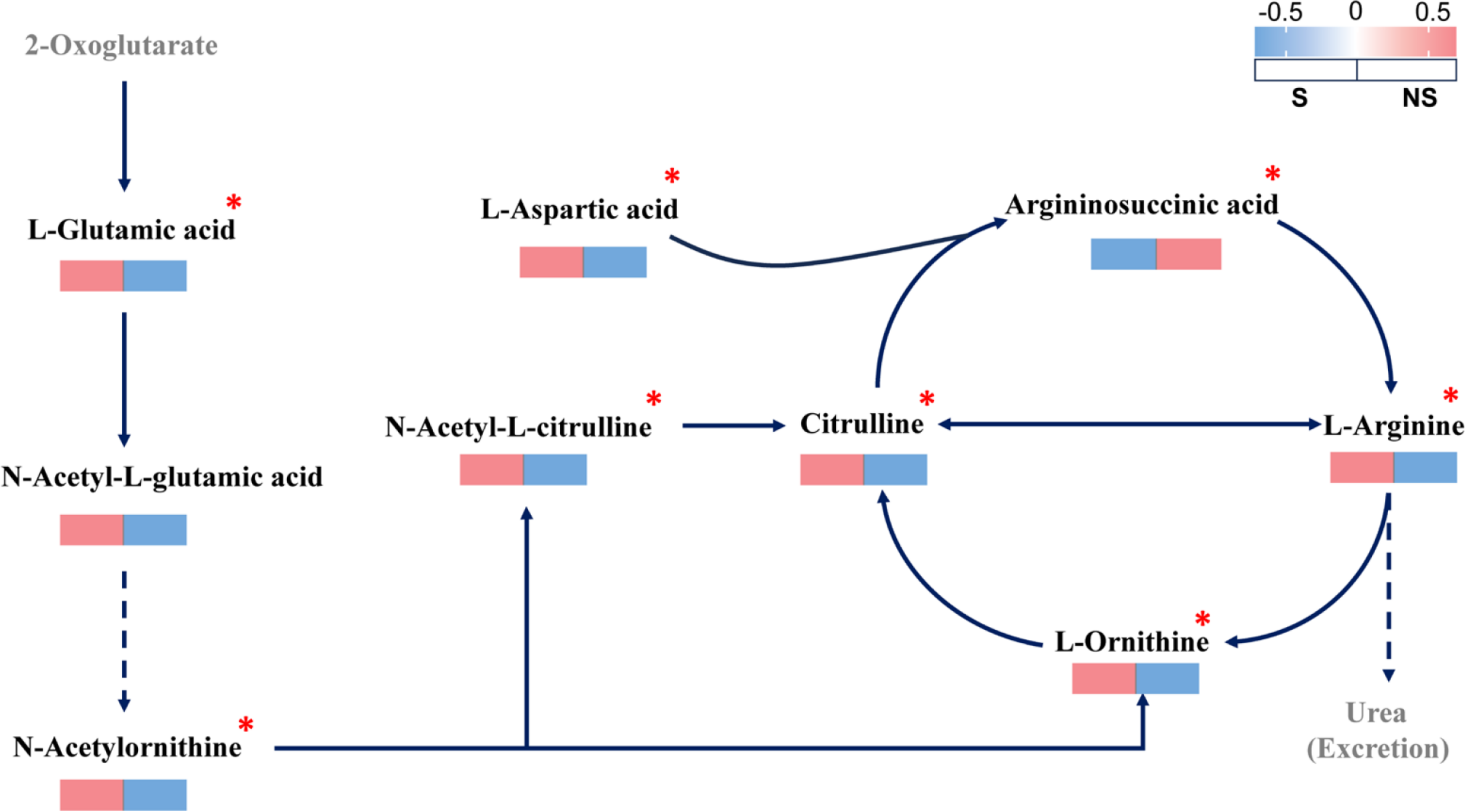
Schematic diagram of the arginine biosynthesis pathway. Solid lines in the figure represent direct upstream–downstream relationships, and the dashed lines represent non-direct upstream–downstream relationships. Gray indicates non-detected metabolites, and black indicates detected metabolites. Red boxes represent upregulated metabolites, and blue boxes represent downregulated metabolites. An asterisk (*) indicates significant differences in metabolite expression levels in the S_vs_NS comparison group (p < 0.05). NS, No shading; S, With shading. (n = 8).

In the arginine biosynthesis metabolic pathway (**Figure 4**), shading significantly increased the contents of seven metabolites, namely L-glutamic acid, N-acetylornithine, L-ornithine, citrulline, L-arginine, N-alpha-acetyl-L-citrulline, and L-aspartic acid, and significantly decreased the argininosuccinic acid content. This indicates that shading activated the positive progression of the arginine biosynthesis metabolic pathway in the rhizosphere soil, and the significant increase in L-arginine accumulation enhanced microbial utilization of L-arginine for N release.

### 3.3 Effects of shading on rhizosphere soil microorganisms and the nitrogen cycle

**Figure 5:**
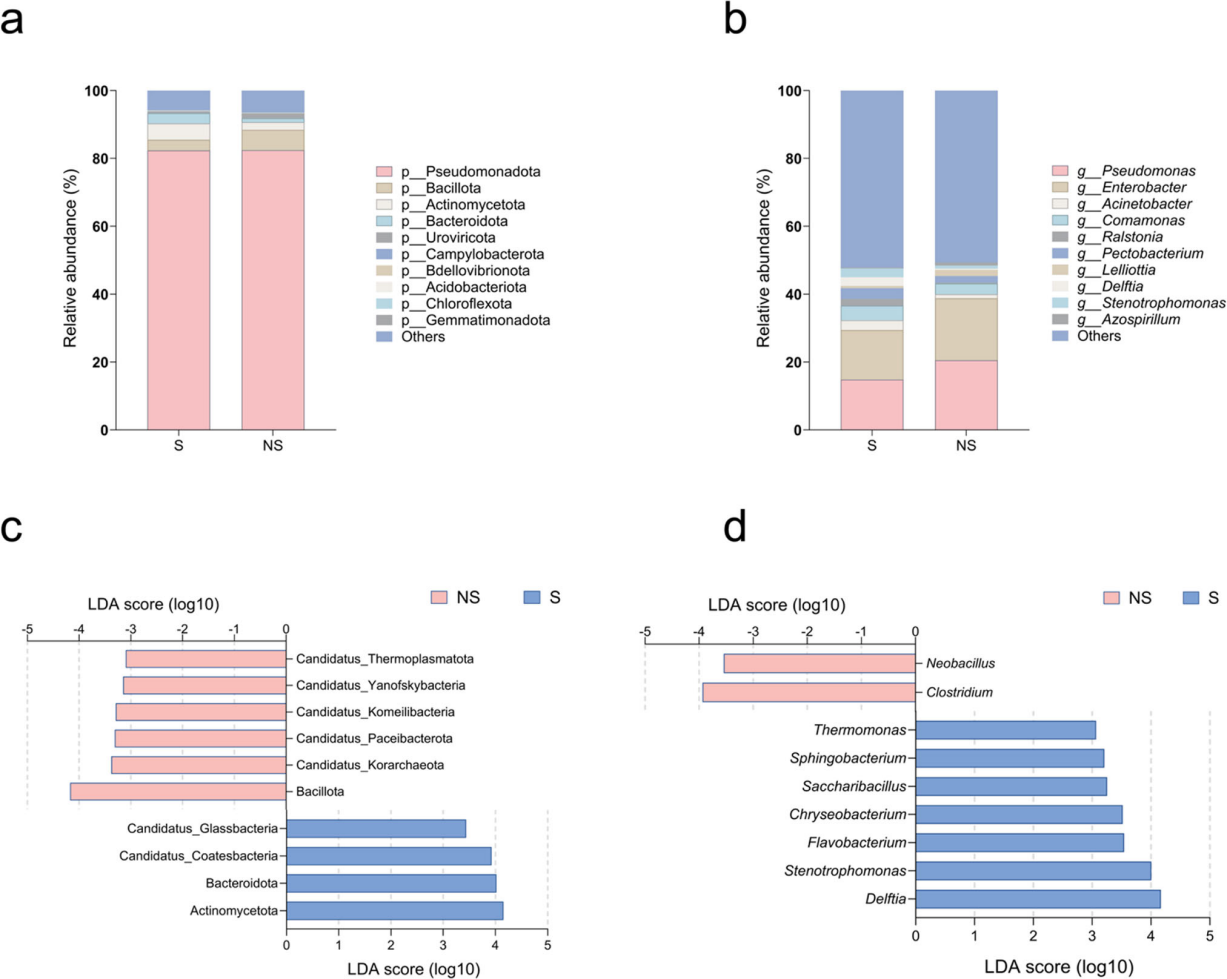
Composition and differential species of the microbial communities in the rhizosphere soil of shaded (S) and non-shaded (NS) tobacco plants (LEfSe analysis, LDA > 3). **(a, b)** Top 10 species ranked by abundance at the phylum and genus levels, respectively. **(c, d)** Differential species at the phylum and genus levels, respectively (n = 8).

Genome-wide analysis revealed that shading affected the species diversity of soil microorganisms in the rhizosphere. At the phylum and genus levels, the Shannon and Simpson indices increased, but the differences did not reach a significant level (*p*>0.05) (**Supplementary Figure S2**). Shading affected the microbial composition in the rhizosphere at the phylum level, with a relative decrease in the abundance of Bacillota and an increase in the relative abundance of Actinomycetota and Bacteroidota (**Figure 5a**). There was a significant increase in both of these dominant species (**Figure 5c**). Shading resulted in significant population differences at the genus level. The abundance of *Pseudomonas* and *Enterobacter* decreased, but that of *Acinetobacter* and *Delftia* increased (**Figure 5b**). *Flavobacterium*, *Stenotrophomonas*, and *Delftia* were all significantly enriched differential species (**Figure 5d**).

**Figure 6:**
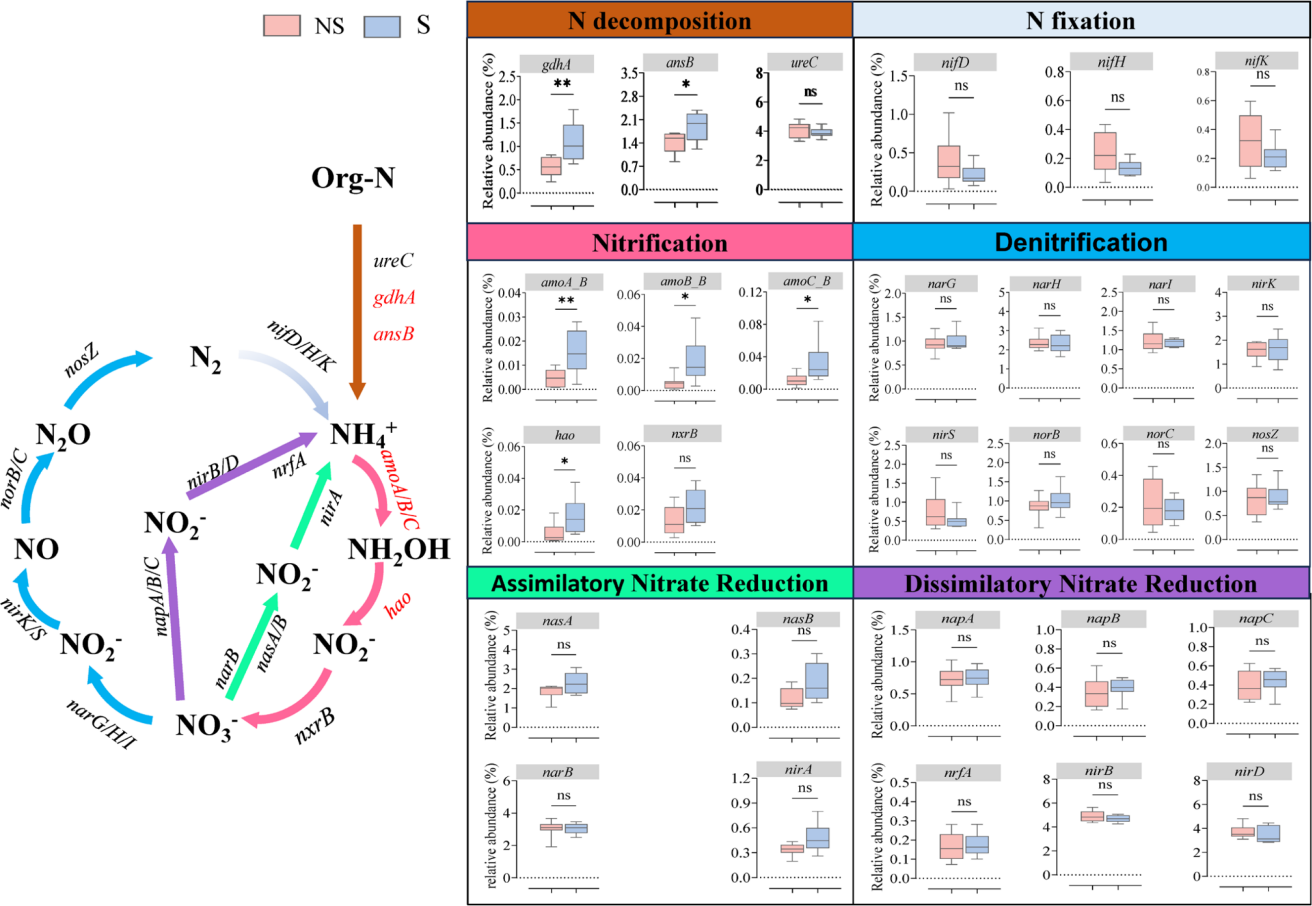
Nitrogen cycle-related functional genes in rhizosphere soil based on metagenomics sequencing. Potential nitrogen fixation process and gene network (gray). Potential nitrogen decomposition process and gene network (brown). Potential nitrification process and gene network (red). Potential denitrification process and gene network (blue). Potential assimilatory nitrate reduction process and gene network (green). Potential dissimilatory nitrate reduction process and gene network (purple). Detailed gene abundance information can be found in **Supplementary Table 6**. Red genes on the lines indicate significant differences in the relative abundance of nitrogen cycle genes between shaded (S) and unshaded (NS) conditions (*p* < 0.05), and the black genes indicate no significant differences (t-test, n = 8) (p > 0.05).

The metagenomics data identified a total of 58 N-cycle functional genes (**Supplementary Table 6**). Based on previous studies (Du et al., 2023; Hu et al., 2025; Lu et al., 2025; Sun et al., 2025b), we selected 29 key functional genes for further analysis (**Figure 6**). Shading significantly increased the relative abundance of six key genes, namely *gdhA*, *ansB*, *amoA_B*, *amoB_B*, *amoC_B*, and *hao.* This indicates that shading has a positive regulatory effect on soil N decomposition and nitrification processes, thus promoting soil N cycling.

**Figure 7:**
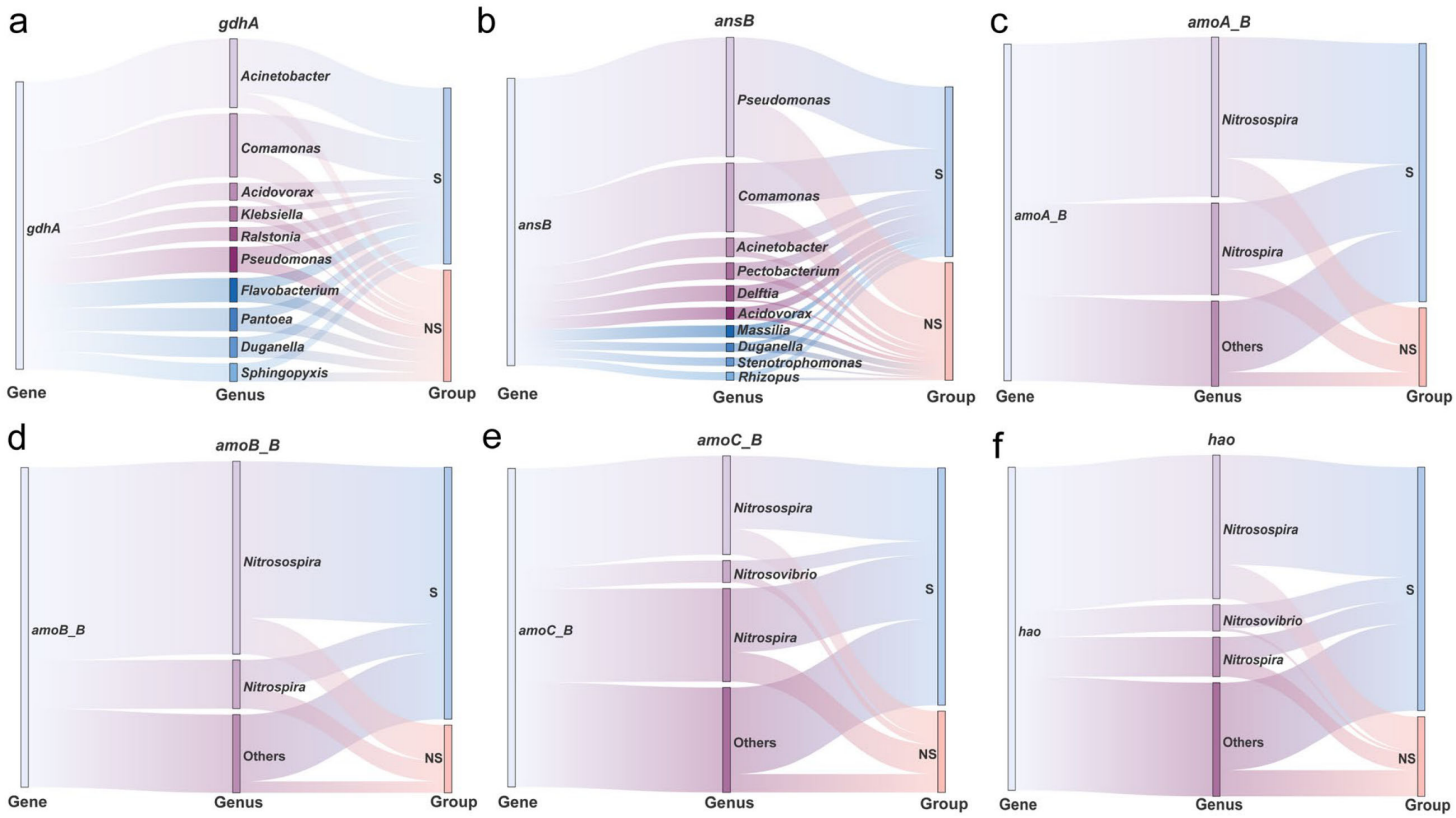
Analysis of key gene species contributions based on the NCycleDB database. Top 10 species contributing to the abundance of **(a)***gdhA*, **(b)***ansB*, **(c)***amoA*_B, **(d)***amoB_B*, **(e)***amoC_B*, and **(f)***hao*. NS, No shading; S, With shading. (n = 8).

To identify the key species corresponding to these six differentially expressed genes (DEGs), we conducted species contribution analysis at the genus level (**Supplementary Table S7**). The 2 N-decomposition DEGs (*gdhA* and *ansB*) shared 15 genera with high contributions: *Acinetobacter*, *Comamonas*, *Acidovorax*, *Klebsiella*, *Ralstonia*, *Pseudomonas*, *Flavobacterium*, *Pantoea*, *Duganella*, *Sphingopyxis*, *Pectobacterium*, *Delftia*, *Massilia*, *Stenotrophomonas*, and *Rhizopus* (**Figures 7a, b**). The 4 nitrification DEGs (*amoA_B*, *amoB_B*, *amoC_B*, *hao*) shared 3 species with high contributions: *Nitrosospira*, *Nitrospira*, and *Nitrosovibrio* (**Figures 7c–f**).

### 3.4 Correlation analysis of differential metabolites with N-cycling species

**Figure 8:**
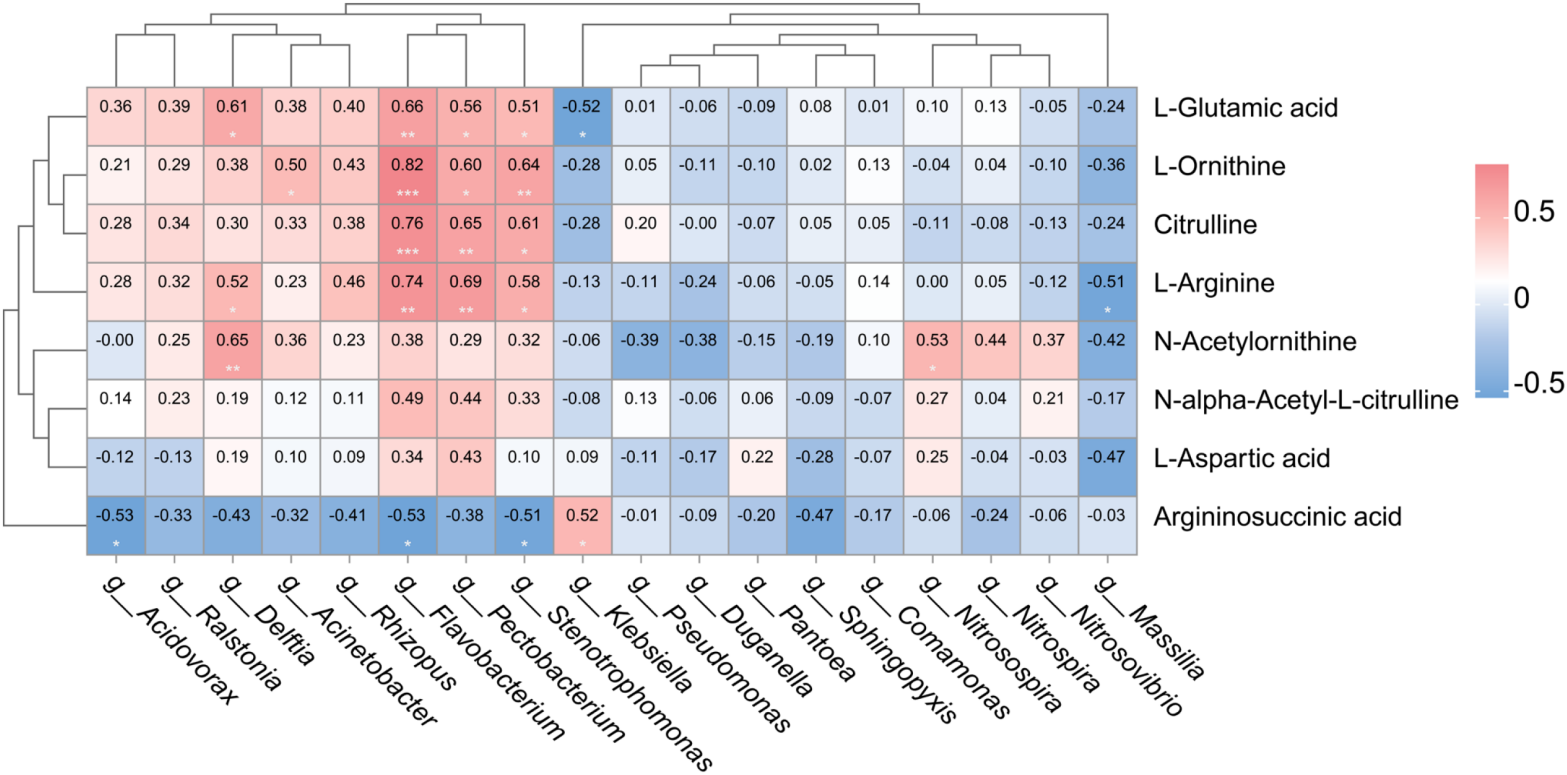
Pearson correlation analysis of differential metabolites and key species in the N cycle. The larger the value in the square, the stronger the correlation. Positive values indicate a positive correlation, and negative values indicate a negative correlation. The marks in the square indicate the significance level of the correlation:*p < 0.05, **p < 0.01, ***p < 0.001 (n = 8).

Correlation analysis was conducted on the 8 key DMAs in the arginine biosynthesis pathway and the 18 N-cycle genera that ranked in the top 10 in terms of contribution (**Figure 8**). L-Glutamic acid, L-ornithine, citrulline, and L-arginine were significantly positively correlated with *Flavobacterium* and *Stenotrophomonas*, but there was no significant correlation between them and *Nitrosospira*, *Nitrospira*, or *Nitrosovibrio*. Argininosuccinic acid was significantly positively correlated with *Klebsiella*, but it was negatively correlated with all other species.

## 4 Discussion

### 4.1 Shading significantly alters the nitrogen absorption characteristics of plants

Light is the core environmental factor regulating the coordinated interaction of carbon (C) and N metabolism in plants. Photosynthetic C assimilation products provide energy (ATP and NADPH) and C skeletons for N absorption and assimilation, directly or indirectly determining the N absorption characteristics of plants and influencing their growth and development processes (Li et al., 2016; Yang et al., 2020; Sathee et al., 2025). In the present study, shading significantly reduced the photosynthetic efficiency and dry matter accumulation of tobacco plants. The chlorophyll content, net photosynthetic rate, and dry matter accumulation decreased significantly (**Figures 1c–e**). Shading reduces the photosynthetic efficiency by inhibiting the synthesis of photosynthetic pigments in the leaves, decreasing the accumulation of photosynthetic products (Yamori et al., 2015; Li et al., 2022; Zhou et al., 2022). Shading resulted in a significant increase in N accumulation in tobacco plants (**Figure 1g**). These results support the phenomenon of “C starvation” in plants caused by shading. To adapt to the adverse effects of low light, plants allocate more photosynthetic substances to their roots, thus enhancing N absorption (Bassiouni et al., 2025). This is regarded as an adaptive strategy to shaded environments because prolonged low light intensity restricts C assimilation in plants, and plants may shift their metabolic focus to N absorption to optimize nutrient investment efficiency (Chen et al., 2024).

### 4.2 Shading significantly affected the characteristics of nitrogen mineralization in the rhizosphere

The rhizosphere, as the crucial interface connecting the plant–soil system, is the core area for nutrient transformation and exchange (Huo et al., 2024; Jia et al., 2025). Research has shown that 30–40% of the photosynthetic products were released into the soil in the form of root exudates, shaping a unique rhizosphere micro-environment (Prescott et al., 2020). During the response to changes in light conditions, plants regulate the composition of their root exudates and have a significant impact on the availability of relevant nutrients in the rhizosphere environment (Martin et al., 2017; Zhao et al., 2021). NH_4_^+^-N and NO_3_^−^-N are the two main forms of N absorbed by plants. The results of the present study indicate that shading significantly increased the SOC, HN, NH_4_^+^-N, and NO_3_^−^-N contents in the rhizosphere soil (**Figure 2**), indicating that shading significantly enhances N availability in the rhizosphere. SOC is a core component of the rhizosphere C-N cycle. Decomposition by microorganisms regulates the accumulation and transformation of NH_4_^+^-N and NO_3_^−^-N (Yang et al., 2024; Dong et al., 2025). Previous studies have shown that shading alters root secretion functions and the composition of plant secretions (Martin et al., 2017). Our results further indicate that shading alters the root exudates released by the plants to the rhizosphere, reshapes the unique rhizosphere microbial community, and changes the N availability in the rhizosphere, a prerequisite for the significant increase in N content in tobacco plants.

Many studies have shown that rhizosphere metabolites are key regulatory factors in the C and N cycles and largely characterize the intensity and direction of nutrient cycling (Coskun et al., 2017; Gong et al., 2023; Lian et al., 2024). In the present study, shading significantly enriched 946 DMAs (**Figure 3b**), among which amino acids, peptides, and analogues accounted for the largest proportion (**Figure 3c**). Amino acids are of vital importance in soil organic N mineralization. Proteins and other N-containing compounds are hydrolyzed into amino acids, which are then utilized by soil microorganisms and deaminated, generating NH_4_^+^-N (Bonde et al., 2001; Geisseler et al., 2010). KEGG pathway enrichment analysis revealed that shading had the most significant impact on arginine biosynthesis in the rhizosphere soil (**Figure 3d**). Arginine is an important component of the soil N reservoir. The most direct result of its mineralization is the rapid release of NH_4_^+^-N, which enhances the N supply potential of the soil (Holik et al., 2017; Chen et al., 2021b). In addition to arginine, four other amino acids (L-glutamic acid, L-ornithine, citrulline, and L-aspartic acid) showed significantly increased expression after shading (**Figure 4**). Studies have shown that when external factors change, plants secrete amino acids through their root systems, regulating their own growth and enhancing their environmental adaptability (Moe, 2013; Ouyang et al., 2025). As important components of soil organic N, amino acids serve as both C and N sources and are directly utilized by soil microorganisms in their intact molecular form or in the form after enzymatic breakdown (Geisseler and Horwath, 2014). Therefore, shading promotes the secretion of various amino acids into the rhizosphere by plants. This enriches the N storage capacity of the soil and the acquisition of microbial N sources and provides sufficient substrates for organic N mineralization, accelerating the transformation of soil N forms and the N cycling process.

### 4.3 Shading significantly affected the structure of the rhizosphere microbial community and the nitrogen cycling process

Soil microorganisms play a crucial role in the N cycle and drive N transformation through various biochemical processes, thereby maintaining the N balance of the ecosystem and providing the N supply necessary for plant growth (Nelson et al., 2016). Changes in external environmental factors directly or indirectly affect the structure and function of soil microbial communities, thereby influencing the N cycle process (Sun et al., 2025a; Zhao et al., 2025). The present study showed that under 30% shading conditions, although there was no significant difference in the diversity of soil microorganisms in the rhizosphere (**Supplementary Figure S2**), the composition of microbial species was altered. The relative abundance of *Flavobacterium*, *Stenotrophomonas*, and *Delftia* significantly increased (**Figure 3d**). *Flavobacterium* is a typical ammonifying bacterium that participates in the decomposition of organic matter in the soil and works with other soil microorganisms to significantly enhance the release of NH_4_^+^ in the soil, directly affecting N availability (Trofymow et al., 1983; Li et al., 2024). Similarly, *Stenotrophomonas* and *Sphingobacterium* have roles in soil N mineralization (Chen et al., 2023; Feng et al., 2023). These results indicate that shading did not cause any changes in the number or diversity of microbial species but selectively enriched soil N cycling functional microorganisms, thus enhancing the conversion of organic N into inorganic N.

Soil microorganisms regulate the soil N cycle by modulating the expression of key genes (Kuypers et al., 2018). In this study, shading significantly increased the abundance of organic N mineralization (*gdhA* and *ansB*) and nitrification process genes (*amoA_B*, *amoB_B*, *amoC_B*, and *hao*), indicating that shading reshaped the soil microbial community structure, regulated the direction and intensity of N cycling, accelerated the N turnover in the rhizosphere soil, and resulted in stronger N mineralization and nitrification. To further clarify the main driving factors behind the N cycle process, we conducted species contribution analysis on N cycle functional genes (**Figure 7**). Genera *Flavobacterium, Stenotrophomonas*, and *Delftia* had a significant contribution to microbial changes, and their relative abundance increased significantly after shading. This further confirmed the key differential genera that drive changes in plant rhizosphere microecology under shading conditions (**Figure 3d**). We also found that *Nitrosospira*, *Nitrospira*, and *Nitrosovibrio* made a significant contribution to functional gene abundance associated with the nitrification process. They are the main ammonia-oxidizing bacteria and play a dominant role in the conversion of NH_4_^+^ to NO_2_^−^ (Zhang et al., 2023; Zhao et al., 2023), indicating that shading also has a promoting effect on soil nitrification. Based on the analysis of regular functional genes, we analyzed the key groups that actually exerted effects using species contribution analysis, which directly verified the accuracy of the previous differential species analysis, providing a new strategy for in-depth exploration of the soil rhizosphere nutrient cycling process based on metagenomics.

### 4.4 Upregulation of the secretion of rhizosphere amino acid substances may be an important factor promoting nitrogen absorption by the plant

We conducted a correlation analysis to reveal the intrinsic relationship among rhizosphere metabolites, microbial communities, and plant N uptake. The enrichment of *Flavobacterium* and *Stenotrophomonas* was significantly positively correlated with L-glutamic acid, L-ornithine, citrulline, and L-arginine accumulation (**Figure 8**). Based on the above analysis, we inferred that tobacco plants under shading conditions may show increased secretion of amino acid substances, recruit relevant microorganisms for organic N degradation, and promote NH_4_^+^-N accumulation. Although the three nitrosation species, *Nitrosospira*, *Nitrospira*, and *Nitrosovibrio*, were not significantly correlated with the amino acid content, an increase in their accumulation promotes the occurrence of nitrification due to the substrate concentration effect of NH_4_^+^-N on nitrification (Booth et al., 2005). Another study found that high N conditions stimulate the activity of nitrifying bacteria (Quan et al., 2016). Therefore, we speculate that the enhanced rate of organic N mineralization is an important factor promoting soil NO_3_^−^-N accumulation. Although shading causes an energy crisis for tobacco plants, they can still utilize root exudates to convert the difficult-to-utilize organic N into easily absorbable inorganic N, promoting N absorption by the tobacco plants and compensating for the growth defects caused by insufficient light. This might be the”cry for help” made by tobacco plants to the soil, as a response to the negative effects on their natural growth caused by the low light environment (Liu, 2025; Liu et al., 2025). Shading inhibits the synthesis of photosynthetic products in tobacco plants. In response, tobacco plants secrete amino acids (arginine, L-glutamic acid, L-ornithine, citrulline, and L-aspartic acid), to recruit functional microorganisms related to the N cycle (*Flavobacterium*, *Stenotrophomonas*, and *Delftia*) and upregulate key genes for N cycling (*gdhA*, *ansB*, *amoA_B*, *amoB_B*, *amoC_B*, and *hao*), promoting the decomposition of soil organic N and nitrification, significantly increasing the soil NH_4_^+^-N and NO_3_^−^-N content, and facilitating N absorption by tobacco plants.

This study revealed the effects of shading on the regulation of soil N cycling in the plant–soil system and the N absorption characteristics of crops, providing a theoretical basis for understanding the mechanism by which crops respond to shading in the rhizosphere. However, this study was conducted during a single growing season, and therefore the generalizability of our findings across different years requires further validation. Within this context, the biological regulatory mechanisms by which shading affects the cycling of other nutrients (such as carbon and phosphorus) in the soil and the responses of related microorganisms to shading remain unclear. Furthermore, under shaded conditions, the interactions between soil nutrients and the modulatory effects of varying nitrogen gradients on plant responses constitute significant subjects for further investigation. Therefore, future studies should incorporate multi-year field trials with different fertilizer gradients, and further utilize combined multi-omics analysis, genetic verification, and other approaches to systematically investigate the interaction between soil biogeochemical processes and plant nutrient feedback under shading. Such efforts will help deepen our understanding of the regulatory functions of rhizosphere microorganisms in plant light adaptation and have significant practical implications for optimizing crop cultivation patterns and field fertilization management through microbial strategies.

## 5 Conclusions

This study utilized metabolomics and metagenomics to determine the changes in rhizosphere metabolic characteristics, microbial community structure, and N cycling processes of tobacco plants under shade cultivation. It revealed the mechanism by which shade affects rhizosphere microorganisms, thereby influencing N absorption in plants at the root-soil interface. Shading significantly inhibits chlorophyll synthesis, the net photosynthetic rate, and the accumulation of photosynthetic products in tobacco plants. Shading alters the metabolic profile of the root system, inducing the synthesis and secretion of amino acids such as L-arginine. These root exudates increase the contents of soil organic carbon (SOC) and organic nitrogen (HN) in the rhizosphere. This altered chemical environment recruits and enriches functional microorganisms involved in nitrogen decomposition and nitrification. Consequently, the release and accumulation of inorganic nitrogen (NH_4_^+^-N and NO_3_^−^-N) are promoted, ultimately leading to a significant increase in the nitrogen content in tobacco plants (**Image 1**). Previous studies have mainly focused on the effects of shading on the aboveground parts of plants, and this study provides a new perspective for understanding how the plant–soil feedback system responds to shading.

## Data Availability

The original contributions presented in the study are included in the article/**Supplementary Material**. Further inquiries can be directed to the corresponding authors.
