# Supplementary material for: Shading affects the nitrogen cycling process and plant nitrogen uptake by altering the rhizosphere microbial community
Source: Front Plant Sci. 2026 Apr 2;17:1780344. doi: 10.3389/fpls.2026.1780344 (PMC13083072; doi:10.3389/fpls.2026.1780344)
Supplement: Supplementary file 3 [file DataSheet1.docx]

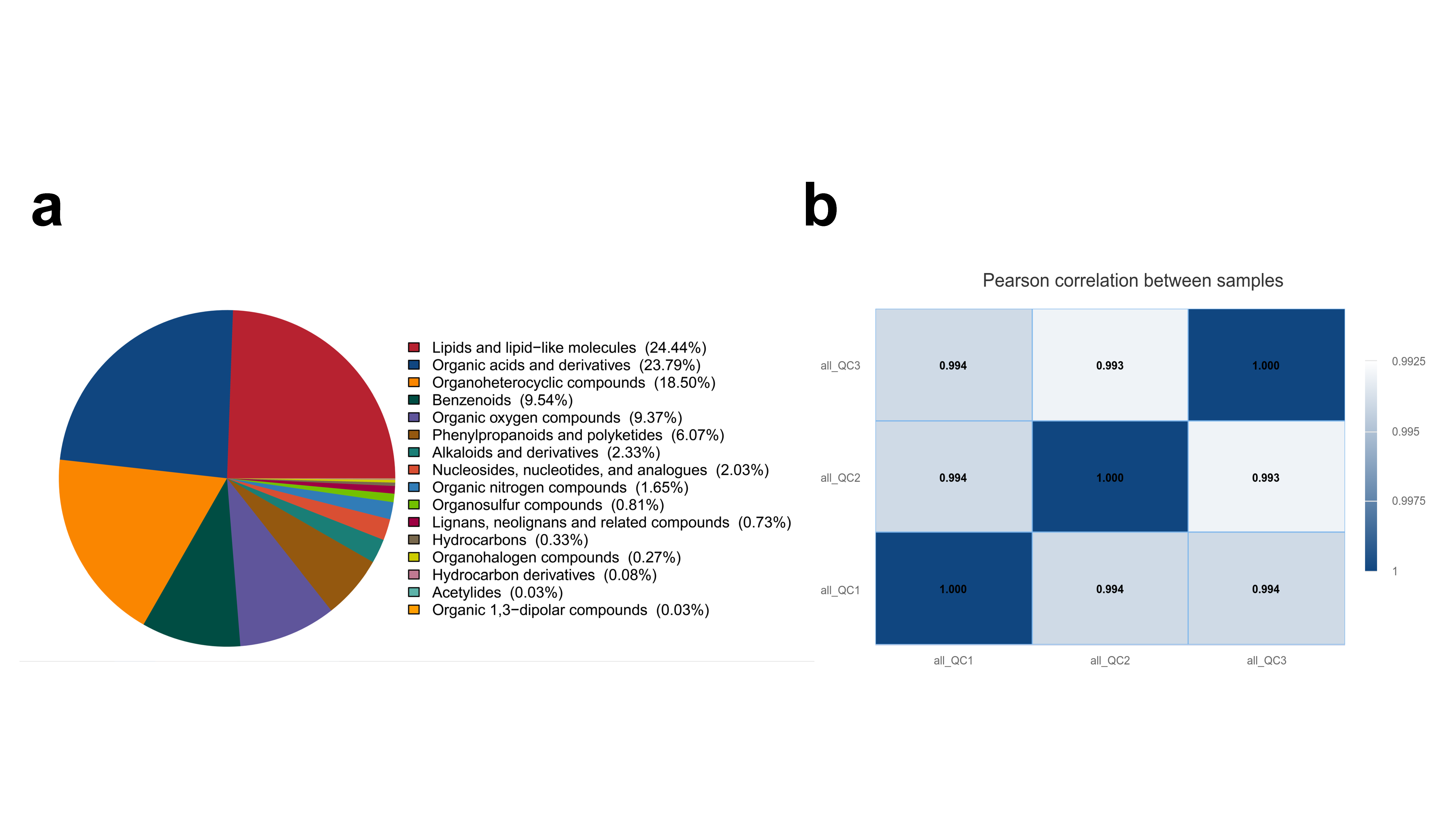


**Figure S1.** Metabolic profiles of rhizosphere soil and quality control sample correlation. (a) Classification of metabolites into the first-level category. (b) Pearson correlation analysis of quality control samples (n = 8).


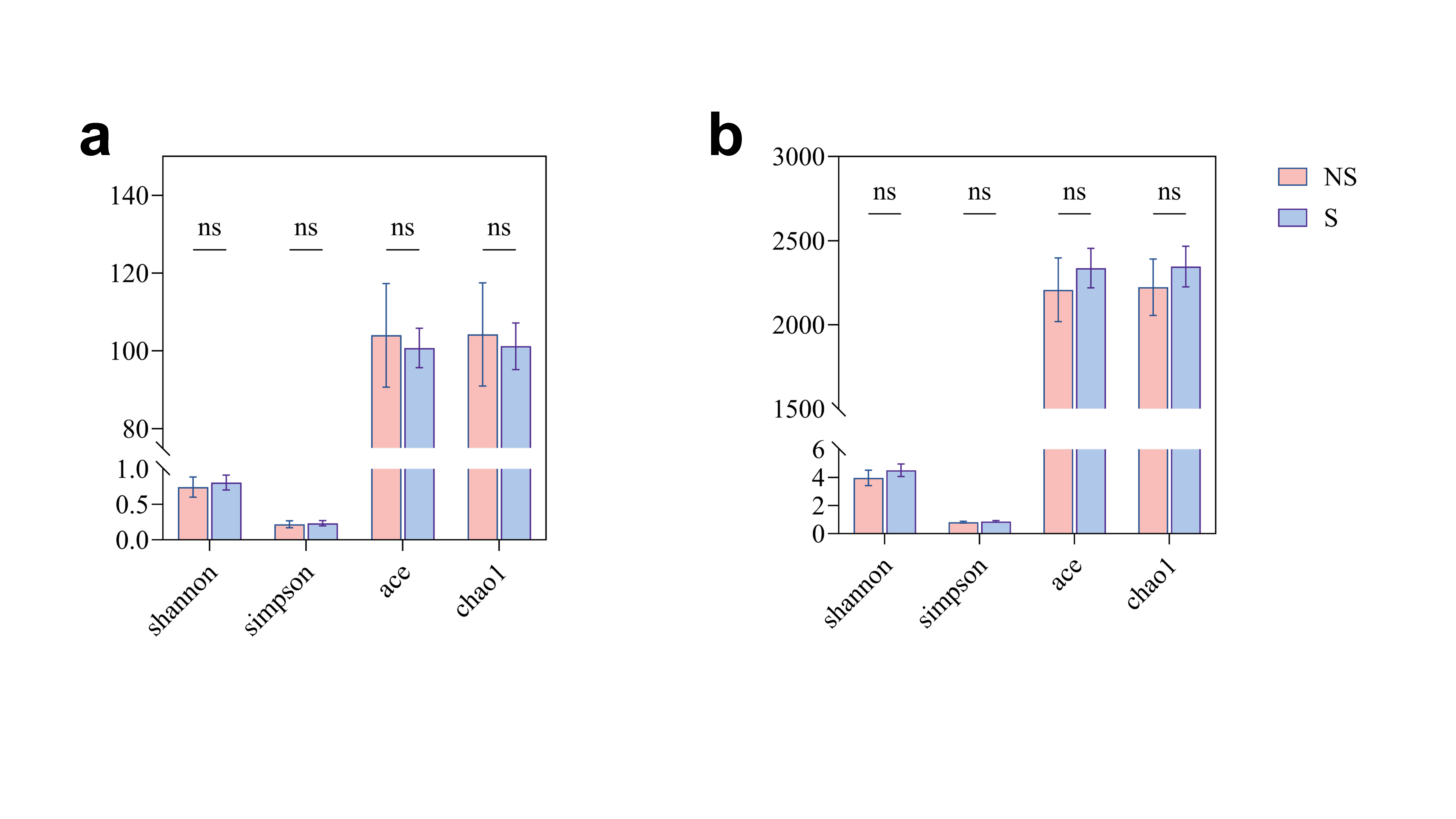


**Figure S2.** Microbial diversity in the rhizosphere soil of shaded (S) and non-shaded (NS) tobacco plants. Diversity index on phylum level (a) and genus level (b). Statistically significant differences are indicated by asterisks (t-test, n = 8): “ns” is not significant. Data are presented as the mean ± standard error.
